# BmTEdb: a collective database of transposable elements in the silkworm genome

**DOI:** 10.1093/database/bat055

**Published:** 2013-07-25

**Authors:** Hong-En Xu, Hua-Hao Zhang, Tian Xia, Min-Jin Han, Yi-Hong Shen, Ze Zhang

**Affiliations:** ^1^State Key Laboratory of Silkworm Genome Biology, The Key Sericultural Laboratory of Agricultural Ministry, Southwest University, Chongqing 400716, China, ^2^Laboratory of Evolutionary and Functional Genomics, School of Life Sciences, Chongqing University, Chongqing 400044, China and ^3^College of Animal Science and Technology, Southwest University, Chongqing 400716, China

## Abstract

The silkworm, *Bombyx mori*, is one of the major insect model organisms, and its draft and fine genome sequences became available in 2004 and 2008, respectively. Transposable elements (TEs) constitute ∼40% of the silkworm genome. To better understand the roles of TEs in organization, structure and evolution of the silkworm genome, we used a combination of *de novo*, structure-based and homology-based approaches for identification of the silkworm TEs and identified 1308 silkworm TE families. These TE families and their classification information were organized into a comprehensive and easy-to-use web-based database, BmTEdb. Users are entitled to browse, search and download the sequences in the database. Sequence analyses such as BLAST, HMMER and EMBOSS GetORF were also provided in BmTEdb. This database will facilitate studies for the silkworm genomics, the TE functions in the silkworm and the comparative analysis of the insect TEs.

**Database URL**: http://gene.cqu.edu.cn/BmTEdb/.

## Introduction

Transposable elements (TEs) are fragments of DNA that can move in a genome and insert themselves into new chromosomal locations (1). They occupy large fractions of higher eukaryotic genomes owing to their ability to increase copy number in the process of transposition. Based on whether the intermediate they use to move is RNA or DNA, eukaryotic TEs have been subdivided into two major classes, class 1 (retrotransposons) and class 2 (transposons) (2). Class 1 TEs use their encoded transcripts (mRNA), not themselves, to form the transposition intermediate to transpose by ‘copy and paste’ mechanism, whereas class 2 TEs transpose via DNA intermediate by the so-called ‘cut-and-paste’ mechanism. Within each class, TEs are further subdivided on the basis of the structural features or enzymatic criteria (3). Class 1 elements are further classified into two subclasses, the elements that are characterized by long terminal repeats (LTR retrotransposons), and the elements that lack long terminal repeats (non-LTR retrotransposons). Autonomous non-LTR retrotransposons (long interspersed nuclear elements, LINEs) are thought to be responsible for transposition of non-autonomous short interspersed nuclear elements (SINEs) (4). Class 2 elements are further classified into three main subclasses, terminal inverted repeats (TIRs), *Helitrons* and *Mavericks* (5).

Although TEs were considered as ‘junk DNA’, now there is compelling evidence that TEs play important roles in the evolution of genes and regulatory networks (6). Besides, the repetitive nature of TEs poses great challenges for genome sequencing, assembly and gene annotation (7, 8). Thus, it is of great importance to identify and annotate TEs in sequenced genomes. However, the identification and classification of TEs in higher eukaryotic genomes are complicated and difficult owing to the fact that their structure and classification are complex, diverse and controversial (3, 9, 10). Nevertheless, a number of approaches and tools reviewed in recent articles (1, 7, 11) have been developed in the past decades. These approaches and tools are divided into three main types: *de novo*, homology-based and structure-based (1, 7). As for *de novo* methods, two basic approaches have been employed—query vs. query similarity searches and word counting/seed extension (1). Because different types of approaches have both advantages and drawbacks, a combined approach is required to accurately identify, classify and annotate TEs in a given genome.

The silkworm, *Bombyx mori*, is one of the major insect model organisms, and its draft genome sequence became available in 2004 (12). TEs constitute ∼40% of the silkworm genome (13). Before the release of the silkworm genome, many TEs had been identified, such as Pao, Kamikaze and Yamato, BMC1, L1Bm, R1Bm, R2Bm, SART1, TRAS1, Bm1 and Mariner. A *de novo* repeat library for silkworm was created using a repeat search program Recovery of Ancestral Sequences (ReAS) (14), which can recover ancestral sequences for TEs from the unassembled whole genome shotgun reads, based on the silkworm whole-genome shotgun sequences (15). And Osanai-Futahashi *et al.* (2008) modified this library by adding 22 known TEs, named it as TELib and annotated the sequences based on homology (13). This library is incomplete because ReAS, like other *de novo* methods, tended to miss or split structurally composite repeats (i.e. LTR retrotransposons, non-LTR retrotransposons) (1). Accordingly, in this work, we used a combined approach to identify, classify and annotate TEs in the silkworm genome, and organized the obtained results into a database, BmTEdb. It can be accessed at the address http://gene.cqu.edu.cn/BmTEdb/.

## Database construction and content

### Data sources

The new assembly of the silkworm genome, the nucleotide sequences of protein-coding genes and the sequences of tRNA and rRNA were downloaded from the Web site of the silkworm genome database, SilkDB v2.0 (16). The silkworm repeat library TELib- and bacterial artificial chromosome (BAC)-assembled sequences were downloaded from the KAIKObase (17). The Repbase Update collection (18), RepBase version 16.04 was downloaded from the Genetic Information Research Institute (http://www.girinst.org/).

## Collection and identification of TEs in the silkworm genome

**Figure 1.**
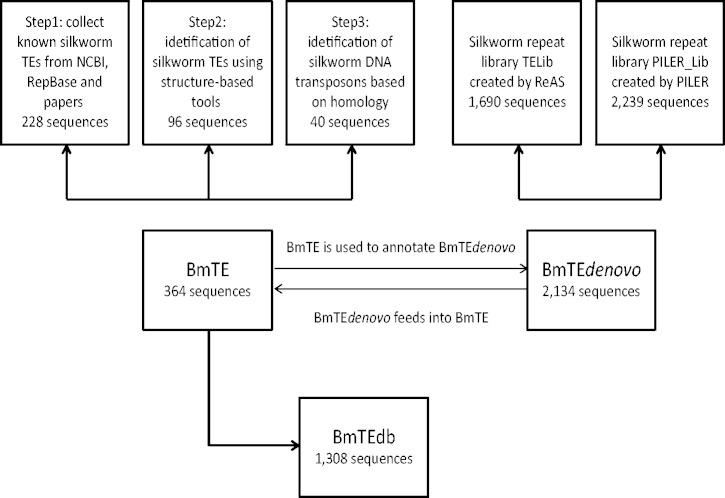
An outline of the collection and identification of TEs in the silkworm genome.

Two libraries are generated in this section: BmTE and BmTE*denovo*. BmTE integrated the results obtained from Step1 to Step 3. BmTE*denovo* is composed of sequences from library generated by PILER (PILER_Lib) and TELib. Because sequences in the former are highly reliable, BmTE is used to annotate the latter one. A flowchart is shown in Figure 1.

Step 1: On 5 June 2011, the NCBI nucleotide database was searched using the following search expression (silkworm [Organism] OR *Bombyx* [Organism]) AND ((((((transposon [All Fields]) OR retrotransposon [All Fields]) OR LTR retrotransposon [All Fields]) OR non-LTR retrotransposon [All Fields]) OR mobile element [All Fields]) OR retro-element [All Fields]). Four hundred eighty sequences were retrieved. Not all these sequences are real silkworm TEs and some of these sequences only include partial regions of TEs. Then the sequences were inspected one by one to check whether it is real TE or to extract TE sequences from the position information provided in the descriptive text. In this step, we excluded 20 sequences. Eighty-seven silkworm TE families were extracted from RepBase version 16.04. Then TE sequences from NCBI were integrated into BmTE after being masked by 87 RepBase-derived TE sequences using RepeatMasker (Smit, AFA, Hubley, R & Green, P. RepeatMasker Open-3.0.1996-2010 <http://www.repeatmasker.org>). Recently, 17 families of miniature inverted transposable elements (MITEs) have been identified in the silkworm genome by a MITE search program named MUST, followed by a strict approach to filter pseudo-MITEs [see (19) for details]. In addition, two families of silkworm SINEs named BmSE and BmSer_SINE have been identified in two separate studies (20, 21). To our knowledge, these TE families have not been submitted to the NCBI nucleotide database, and thus we collected these families into our data set. Besides, 22 families of Tc1/Mariner DNA transposons in the silkworm genome have been identified using the same methods for DNA transposons in Step 3 (Zhang HH, unpublished data), and these consensus sequences were also included. In this step, 228 sequences were generated (Figure 1).

Step 2: Structure-based computational tools LTR_STRUC (22), LTR_FINDER (23) and MGEScan-non-LTR (24) with default parameters were used to search the new assembly of the silkworm genome (15), the silkworm repeat library TELib and BAC assembled sequences to identify the LTR retrotransposons and non-LTR retrotransposons. LTR retrotransposons identified from the draft sequence of the silkworm genome were included in this study (25). For LTR retrotransposons, the obtained candidates with known silkworm transposons in their LTRs were removed. Then LTR retrotransposons were searched for known pfam models online. We considered LTR retrotransposons with similarity to these pfam models [Peptidase_A17 (PF05380), RVT_1 (PF00078), RVT_2 (PF07727), Retrotransposon gag protein (PF03732), Integrase DNA binding domain (PF00552), Integrase core domain (PF00665), Retroviral aspartyl protease (PF00077 and PF08284), zf-H2C2 (PF09337), DUF1759 (PF03564) and DUF1758 (PF05585)] as full-length LTR retrotransposons. The cut-off e-value for this process is 1 e-5. Because these full-length LTR retrotransposons have few full-length copies (<3) in the silkworm genome, we cannot identify consensus sequences. And for each family, we keep a full-length LTR retrotransposon as a representative. For non-LTR retrotransposons, obtained non-LTR retrotransposons were first masked by known non-LTR retrotransposons from RepBase using RepeatMasker. Non-LTR retrotransposons of which >80% were masked were removed, and new candidates were used as queries to search silkworm genomes. The consensus was created if there were more than three copies; otherwise the candidates were retained as a representative. In this step, 92 LTR retrotransposons families and 4 non-LTR retrotransposons families were identified (Figure 1).

Step 3: Two hundred thirty-eight transposase protein sequences from insect DNA transposons were extracted from the RepBase version 16.04. These sequences were used as queries for tBLASTN search against the new assembly of the silkworm genome. The sequences whose coverage was <50% or whose similarity to the query was <30% were removed. The obtained sequences with 5 kb of flanking region were searched by using FastPCR v6.1 (26) to look for TIRs with initial searching word size of 12, filter minimal string length of 17, minimal alignment string length of 18, gap size between strings of 10, local similarity of 75 and alignment output length of 100. In total, 40 DNA families were identified in this step (Figure 1).

The above results were integrated into a silkworm TE library, BmTE, and the redundant sequences were removed from the library based on the standard used in the previous study (27). Specifically, sequences within the library were compared with one another using BLASTN, and any sequences covering ≥90% of the length of, and with ≥90% identity to, any other sequences were discarded. This library was used to annotate the following repetitive sequences identified using *de novo* approaches because these sequences were highly reliable (7).

The *de novo* program PILER-DF (28) was used to identify repetitive sequences in the new assembly of the silkworm genome. It relies on the pairwise aligner for long sequences (PALS) algorithm that finds local alignments between DNA sequences by aligning the genome to itself. Because the genome is too large to align the entire sequence to itself, we assigned the scaffolds into chromosomes according to the mapping information indicated in the SilkDB v2.0 (16). After this, PALS was used to align one chromosome to another. The minimum hit length is specified by the -length option (200 in this study), the minimum identity by the -pctid option (94.0 used in this study).

The number of sequences in the PILER_Lib is 2239. PILER_Lib was integrated with TELib [total 1690 sequences, 1668 ReAS *de novo* repetitive sequences, 17 known TEs and five sequences contributed by Hiroaki Abe (13)], and the resulting library was designated as BmTE*denovo*. Sequences in the BmTE*denovo* were sorted by length from short to long, and the redundant sequences were removed from BmTE*denovo* based on the above standard. The non-redundant 2220 sequences were checked to rule out two potential types of false positives: sequences with close similarity to the annotated genes (protein-coding genes, tRNA and rRNA) in the silkworm, and sequences containing a significant fraction (>25%) of tandem repeats, as determined by Tandem Repeat Finder (TRF) (29) with recommended parameters on the TRF Web site (http://tandem.bu.edu/trf/trf.unix.help.html). After this procedure, 2134 TE sequences were obtained.

### Classification and annotation of TEs in the silkworm genome

The resulting 2134 sequences were further analyzed for their TE class. Specifically, TE sequences with ≥90% nucleic acid identity as well as >50% in length to any sequences in the library BmTE were discarded from BmTE*denovo*. For remaining sequences, tBLASTX searches were performed by CENSOR (30) in the sensitive mode using BmTE and RepBase16.04 as repeat libraries. The sequences with >50% similarity were removed from BmTE*denovo* and added into BmTE. The definition of ‘Chimera’ and ‘Others’ was based on the criteria used in the previous study (13). The rest of the sequences were classified by using TEclass (31), a tool classifying unknown TEs into their functional categories using machine learning support vector machine for classiﬁcation. These sequences were classified as ‘unknown’.

## False-positive rate of this pipeline

We tested LTR_STRUC and MGEScan-non-LTR using reversed silkworm genomic sequence. LTR_STRUC returned 350 LTR retrotransposons candidates. And after a validation of known silkworm TEs in LTRs of LTR retrotransposons candidates, 350 sequences were retained. Then we considered LTR retrotransposons with similarity to these pfam models (see above) as full-length LTR retrotransposons. Only one candidate was retained, and the e-value was 0.035, larger than the cutoff of 1 e-5. MGEScan-non-LTR returned no results using reversed silkworm genomic sequence. So the false discovery rate of LTR_STRUC and MGEScan-non-LTR plus additional process is estimated to be 0.

We also tested PALS (search engine) and PILER using shuffled silkworm genomic sequences. Each silkworm scaffold sequence was shuffled with the entropy source of TRUE_RANDOM and the starting ordinal of zero by shuffle program, which was downloaded from http://eyegene.ophthy.med.umich.edu/shuffle/. PALS did not find any local alignments between these shuffled sequences. Thus, the false discovery rate of PALS and PILER is estimated to be 0.

## Results and user interface

Using the approaches above, we identified 1308 TE families in the silkworm genome and organized these families and their classification information into an easy-to-use web-based database, BmTEdb. The composition of BmTEdb can be found in Table 1. The BmTEdb web interface is organized into four functional sections, data browsing section, keywords search section, sequences analysis tools section and help information section.

**Table 1.** Description of TEs deposited in BmTEdb

Class	Order	Superfamily	Number of entries
Class 1	LTR	*Copia*	23
		*Gypsy*	121
		*Bel*	73
		Chimera LTR	3
		Unknown LTR	119
	LINE	*CR1*	22
		*CRE*	4
		*Daphne*	18
		*I*	27
		*Jockey*	14
		*Kiri*	1
		*L1*	7
		*L2*	12
		*LOA*	1
		*Nimb*	1
		*Proto2*	4
		*R1*	32
		*R2*	1
		*R4*	8
		*RTE*	41
		*Vingi*	2
		*Chimera LINE*	11
		*Unknown LINE*	264
	SINE	*BM1*	3
		*BM1-related*	5
		*BmSer*	1
		*tRNA*	2
		*Unknown SINE*	6
Class 2	TIR	*Academ*	4
		*Chapaev*	4
		*EnSpm*	1
		*Harbinger*	9
		*hAT*	11
		*ISL2EU*	1
		*Tc1/Mariner*	32
		*P*	6
		*piggyBac*	15
		*Sola*	21
		Unknown TIR	269
		*Transib*	3
		*Zator*	1
	MITE	MITE	19
	Helitron	*Helitron*	7
Other	Other	Other	79

**Figure 2.**
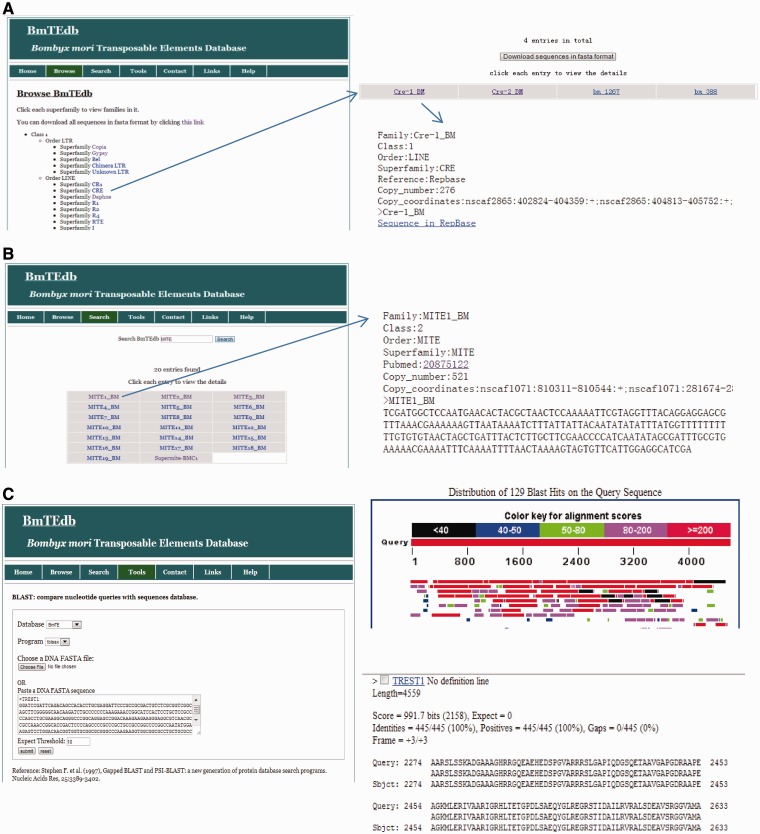
Different functional sections of BmTEdb web interface. (**A**) The browsing interface of database BmTEdb. All TE sequences in BmTEdb were classified into different classes, orders and superfamilies. Users may choose superfamily they are interested to view the families that belong to the chosen TE superfamily. The detailed information of each family can be retrieved by clicking the corresponding entry, including the classification information, references, nucleotide sequences, copy numbers and coordinates of each copy. (**B**) The searching interface of database BmTEdb. Users can search for TE sequences with keywords through the keyword search interface. (**C**) The BLAST interface of database BmTEdb. A sample of tBLASTN results was shown.

In the browsing interface, classification structures of TEs in BmTEdb are shown. Users can browse different superfamilies by clicking them, as shown in Figure 2A. And the detailed information of each family in this superfamily can be retrieved by clicking the corresponding entry, including the classification information, references, nucleotide sequences, copy numbers and coordinates of each copy (Figure 2A).

In the keyword search interface of BmTEdb, users can use a keyword to search the BmTEdb (TE class, TE order, TE superfamily, TE family and references) to find entries interesting to the users, as shown in Figure 2B.

The web interface of sequence analysis tools is provided to facilitate the quick comparison of users’ sequences with the silkworm TEs deposited in BmTEdb. Three types of tools, BLAST (32), GetORF [EMBOSS (33)] and HMMER (34) are included in the BmTEdb infrastructure to assist the annotation of TE elements based on nucleotide sequence and protein sequence. Through this interface, users can submit the query sequences to do BLASTN or tBLASTN against the BmTEdb for homology search. Users can also search the potential open reading frame (ORF) of the query sequence in the GetORF page, and then search protein sequences against TE profile-HMM (profile hidden Morkov model) database collected from previous studies (24, 35). In them, these models were used in the identification of protein domains in LTR retrotransposons and the classification of non-LTR retrotransposons superfamilies. HMMER is provided to facilitate the identification and classification of TEs from evolutionarily distant species that do not show similarity to silkworm TEs at nucleotide level. A sample of tBLASTN results is shown in Figure 2C.

In the help information section, a user’s manual is included in the interface to help the users to learn how to use BmTEdb. Besides the help section, a collection of computational resources for TEs is provided in the links page.

## Conclusion

We have generated a comprehensive TE database for *B. mori*, BmTEdb. This database currently consists of 1308 TE families in the silkworm genome along with classification information. Various web interfaces are provided in support of using BmTEdb by users. One unique feature of BmTEdb is that it allows users to search the potential ORF of the nucleotide sequence, and then search protein sequences against a customized TE profile-HMM database. BmTEdb will be valuable for study of the silkworm TEs. In addition, the availability of the complete set of TEs from *Lepidoptera* species allows evolutionary and comparative analyses of TEs between *Lepidoptera* and other insect species at the whole genome level.

## Accessibility

The publicly accessible BmTEdb Web site is http://gene.cqu.edu.cn/BmTEdb/. All data deposited in the database except the data that derive directly from RepBase are freely available to all users without any restrictions.
